# Crystal structure of (1*S*,2*R*,4*R*,9*S*,11*S*,12*R*)-9α-hy­droxy-4,8-dimethyl-12-[(thio­morpholin-4-yl)meth­yl]-3,14-dioxatri­cyclo­[9.3.0.0^2,4^]tetra­dec-7-en-13-one

**DOI:** 10.1107/S205698901500170X

**Published:** 2015-01-31

**Authors:** Ahmed Benharref, Mohamed Akssira, Lahcen El Ammari, Mohamed Saadi, Moha Berraho

**Affiliations:** a Laboratoire de Chimie des Substances Naturelles, URAC16, Faculté des Sciences Semlalia, BP 2390 Bd My Abdellah, 40000 Marrakech, Morocco; b Laboratoire de Chimie Bioorganique et Analytique, URAC 22, BP 146, FSTM, Université Hassan II, Mohammedia-Casablanca 20810 Mohammedia, Morocco; c Laboratoire de Chimie du Solide Appliquée, Faculté des Sciences, Avenue Ibn Battouta BP 1014, Rabat, Morocco

**Keywords:** crystal structure, *Anvillea radiata*, medicinal compound, hydrogen bonding

## Abstract

The title compound, C_19_H_29_NO_4_S, was synthesised from 9α-hy­droxy­parthenolide (9α-hy­droxy-4,8-dimethyl-12-methyl­ene-3,14-dioxatri­cyclo­[9.3.0.0^2,4^]tetra­dec-7-en-13-one), which was isolated from the chloro­form extract of the aerial parts of the plant *Anvillea radiata*. The mol­ecule is built up from two fused five- and ten-membered rings, with an additional ep­oxy ring system and a thio­morpholine group as a substituent. The ten-membered ring adopts an approximate chair–chair conformation, while the thio­morpholine ring displays a chair conformation and the five-membered ring has an envelope conformation, with the C atom closest to the hy­droxy group forming the flap. An intra­molecular O—H⋯N hydrogen bond closes an *S*(8) ring. The crystal structure features weak C—H⋯O hydrogen-bonding inter­actions, which link the mol­ecules into [010] chains.

## Related literature   

For background to the medicinal uses of the plant *Anvillea radiata*, see: El Hassany *et al.* (2004 ▸); Abdel Sattar *et al.* (1996 ▸). For the reactivity of this sesquiterpene, see: Hwang *et al.* (2006 ▸); Neelakantan *et al.* (2009 ▸); Loubidi *et al.* (2014 ▸).
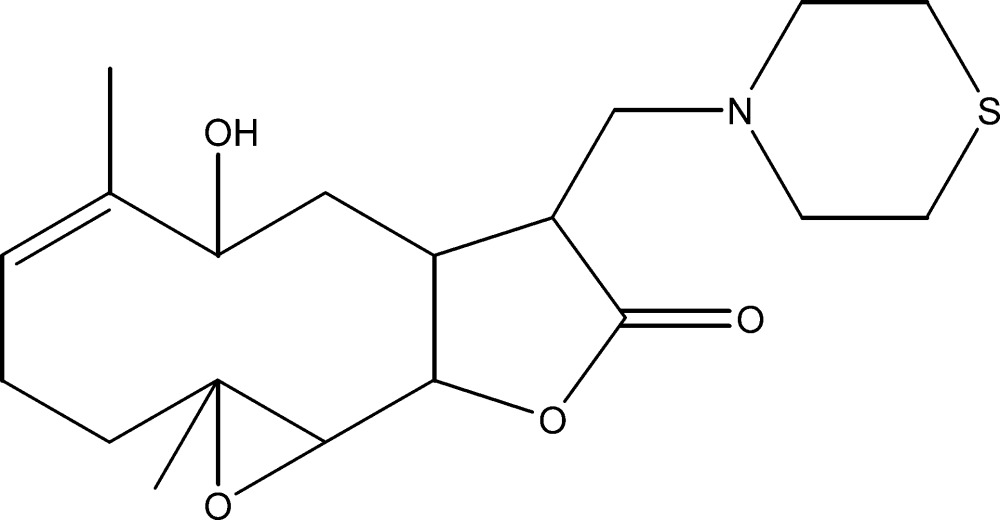


## Experimental   

### Crystal data  

C_19_H_29_NO_4_S
*M* *_r_* = 367.49
Monoclinic, 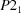

*a* = 11.920 (2) Å
*b* = 6.7919 (13) Å
*c* = 12.144 (3) Å
β = 101.659 (6)°
*V* = 962.9 (3) Å^3^
*Z* = 2
Mo *K*α radiation
μ = 0.19 mm^−1^
*T* = 296 K
0.33 × 0.17 × 0.04 mm

### Data collection  

Bruker APEXII CCD diffractometer
12288 measured reflections
3940 independent reflections
3749 reflections with *I* > 2σ(*I*)
*R*_int_ = 0.033

### Refinement  

*R*[*F* ^2^ > 2σ(*F* ^2^)] = 0.031
*wR*(*F* ^2^) = 0.083
*S* = 1.03
3940 reflections
229 parameters
1 restraint
H-atom parameters constrained
Δρ_max_ = 0.23 e Å^−3^
Δρ_min_ = −0.18 e Å^−3^
Absolute structure: Flack & Bernardinelli (2000 ▸), 1799 Friedel pairs
Absolute structure parameter: 0.04 (7)

### 

Data collection: *APEX2* (Bruker, 2009 ▸); cell refinement: *SAINT-Plus* (Bruker, 2009 ▸); data reduction: *SAINT-Plus*; program(s) used to solve structure: *SHELXS97* (Sheldrick, 2008 ▸); program(s) used to refine structure: *SHELXL97* (Sheldrick, 2008 ▸); molecular graphics: *ORTEP-3 for Windows* (Farrugia, 2012 ▸) and *PLATON* (Spek, 2009 ▸); software used to prepare material for publication: *WinGX* (Farrugia, 2012 ▸).

## Supplementary Material

Crystal structure: contains datablock(s) I, global. DOI: 10.1107/S205698901500170X/hb7355sup1.cif

Structure factors: contains datablock(s) I. DOI: 10.1107/S205698901500170X/hb7355Isup2.hkl

Supporting information file. DOI: 10.1107/S205698901500170X/hb7355Isup3.cml

**Fig. 1.**
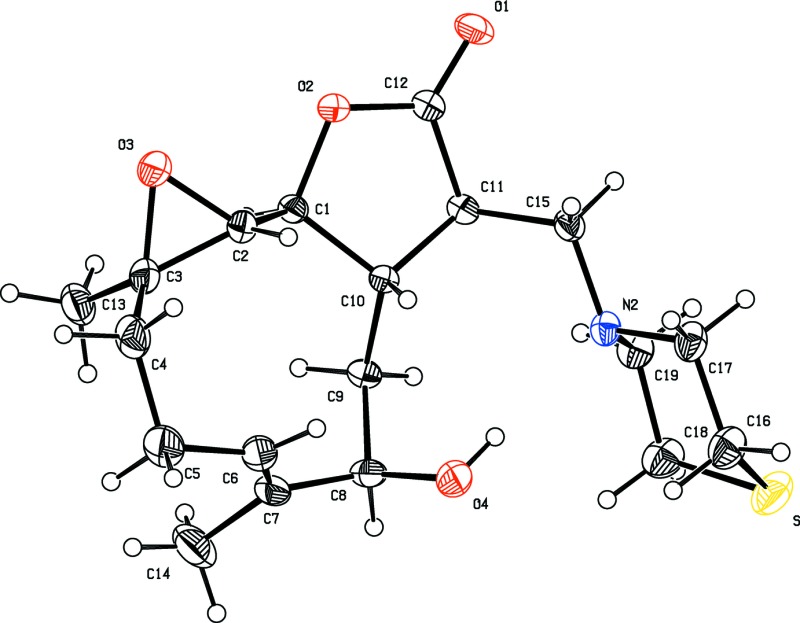
: Molecular structure of the title compound with displacement ellipsoids drawn at the 30% probability level.

**Fig. 2.**
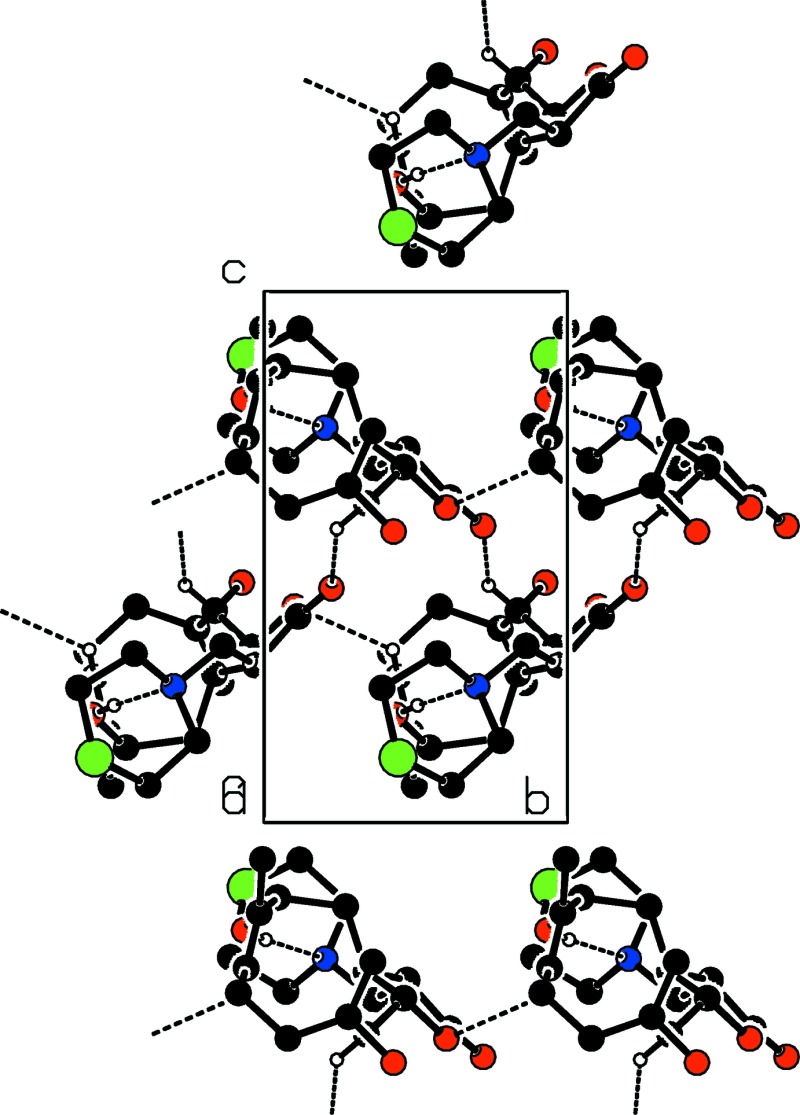
: Partial packing view showing the C—H···O interactions (dashed lines) and the formation of a chain parallel to the b axis. H atoms not involved in hydrogen bonding have been omitted for clarity. [Symmetry code: (i) x, -1 + y, z.]

. DOI: 10.1107/S205698901500170X/hb7355fig1.tif
Mol­ecular structure of the title compound with displacement ellipsoids drawn at the 30% probability level.

b x y z . DOI: 10.1107/S205698901500170X/hb7355fig2.tif
Partial packing view showing the C—H⋯O inter­actions (dashed lines) and the formation of a chain parallel to the *b* axis. H atoms not involved in hydrogen bonding have been omitted for clarity. [Symmetry code: (i) *x*, −1 + *y*, *z*.]

CCDC reference: 1045641

Additional supporting information:  crystallographic information; 3D view; checkCIF report

## Figures and Tables

**Table 1:** Hydrogen-bond geometry (, )

*D*H*A*	*D*H	H*A*	*D* *A*	*D*H*A*
O4H4N2	0.82	2.21	3.0278(18)	176
C2H2O1^i^	0.98	2.51	3.2948(19)	137
C6H6O2^ii^	0.93	2.59	3.2526(19)	129

Symmetry codes: (i) 
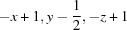
; (ii) 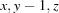.

## supplementary crystallographic information

### S1. Comment

The natural sesquiterpene lactone, 9α -hydroxypartenolide is the main constituent of the chloroform extract of the aerial parts of Anvillea radiata (El Hassany et al., 2004; Qureshi et al.) and of Anvillea garcini (Abdel Sattar et al.,1996). The reactivity of this sesquiterpene lactone and its derivatives have been the subject of several studies (Hwang et al., 2006; Neelakantan et al., 2009; Loubidi et al., 2014), in order to prepare products with high value which can be used in the pharmacological industry. In this context, we have treated the 9α-hydroxy-parthenolide with an equivalent amount of thiomorpholine and prepared the 9α-Hydroxy-12- [thiomorpholin-N- methyl]-4,8-dimethyl-3,14-dioxatricyclo [9.3.0.0^2^,^4^] tetradec-7-en-13-one. The structure of this new product was confirmed by its single crystal X-ray structure. The molecule contains a fused ring system and thiomorpholin group as a substituent to a lactone ring. The molecular structure of (I), Fig.1, shows the lactone ring to adopt an envelope conformation, as indicated by puckering parameters Q = 0.2241 (15) Å and φ = 291.3 (4)°. The atom C10 deviate from the mean plane through other four atoms in the ring by 0.3601 (13)Å. The ten-membered ring displays an approximate chair-chair conformation, while the thiomorpholin ring has an almost ideal chair conformation with QT = 0.6400 (17)Å, θ = 178.37 (15)° and φ = 244 (4)°. In the crystal structure, the molecules are linked by C—H···O intermolecular hydrogen bonds into chains along the b axis (Table 1, Fig.2). In addition an intramolecular O—H···N, hydrogen bond is also observed.

### S2. Experimental

The mixture of 9α-hydoxypartenolide (9α-hydroxy-4,8-dimethyl- 12-methylene- 3,14-dioxatricyclo[9.3.0.0^2^,^4^]tetradec-7-en-13-one) (1 g, 3.8 mmol) and one equivalent of thiomorpholin in EtOH (20 ml) was stirred for ten hours at room temperature. Then the reaction was stopped by adding water (10 ml) and the solution was extracted with chloroform (3 *x* 20 ml). The combined organic layers were dried over anhydrous MgSO_4_, filtered and concentrated under vacuum to give 0.9 g(2.5 mmol) of the title compound (yield: 66%). Recrystallization was performed from ethyl acetate solution to yield colourless plates.

### S3. Refinement

All H atoms were fixed geometrically and treated as riding with C—H = 0.96 Å (methyl),0.97 Å (methylene), 0.98 Å (methine) with *U*_iso_(H) = 1.2Ueq(methylene, methine and OH) or *U*_iso_(H) = 1.5Ueq(methyl). Owing to the presence of S atom, the absolute configuration could be fully confirmed, by refining the Flack parameter (Flack & Bernardinelli (2000) as C1(S), C2(*R*), C4(*R*), C9(S), C11(S) and C2(*R*).

### Crystal data

**Table:**

C_19_H_29_NO_4_S	*F*(000) = 396
*M**_r_* = 367.49	*D*_x_ = 1.268 Mg m^−^^3^
Monoclinic, *P*2_1_	Mo *K*α radiation, λ = 0.71073 Å
Hall symbol: P 2yb	Cell parameters from 3940 reflections
*a* = 11.920 (2) Å	θ = 2.7–26.4°
*b* = 6.7919 (13) Å	µ = 0.19 mm^−^^1^
*c* = 12.144 (3) Å	*T* = 296 K
β = 101.659 (6)°	Platelet, colourless
*V* = 962.9 (3) Å^3^	0.33 × 0.17 × 0.04 mm
*Z* = 2	

### Data collection

**Table:**

Bruker APEXII CCD diffractometer	3749 reflections with *I* > 2σ(*I*)
Radiation source: fine-focus sealed tube	*R*_int_ = 0.033
Graphite monochromator	θ_max_ = 26.4°, θ_min_ = 2.7°
ω and φ scans	*h* = −14→14
12288 measured reflections	*k* = −8→8
3940 independent reflections	*l* = −15→15

### Refinement

**Table:**

Refinement on *F*^2^	Secondary atom site location: difference Fourier map
Least-squares matrix: full	Hydrogen site location: inferred from neighbouring sites
*R*[*F*^2^ > 2σ(*F*^2^)] = 0.031	H-atom parameters constrained
*wR*(*F*^2^) = 0.083	*w* = 1/[σ^2^(*F*_o_^2^) + (0.0458*P*)^2^ + 0.1195*P*] where *P* = (*F*_o_^2^ + 2*F*_c_^2^)/3
*S* = 1.03	(Δ/σ)_max_ < 0.001
3940 reflections	Δρ_max_ = 0.23 e Å^−^^3^
229 parameters	Δρ_min_ = −0.18 e Å^−^^3^
1 restraint	Absolute structure: Flack & Bernardinelli (2000), 1799 Friedel pairs
Primary atom site location: structure-invariant direct methods	Absolute structure parameter: 0.04 (7)

### Special details

**Table:**

Geometry. All e.s.d.'s (except the e.s.d. in the dihedral angle between two l.s. planes) are estimated using the full covariance matrix. The cell e.s.d.'s are taken into account individually in the estimation of e.s.d.'s in distances, angles and torsion angles; correlations between e.s.d.'s in cell parameters are only used when they are defined by crystal symmetry. An approximate (isotropic) treatment of cell e.s.d.'s is used for estimating e.s.d.'s involving l.s. planes.
Refinement. Refinement of *F*^2^ against all reflections. The weighted *R*-factor *wR* and goodness of fit *S* are based on *F*^2^, conventional *R*-factors *R* are based on *F*, with *F* set to zero for negative *F*^2^. The threshold expression of *F*^2^ > 2σ(*F*^2^) is used only for calculating *R*-factors(gt) *etc*. and is not relevant to the choice of reflections for refinement. *R*-factors based on *F*^2^ are statistically about twice as large as those based on *F*, and *R*- factors based on all data will be even larger.

### Fractional atomic coordinates and isotropic or equivalent isotropic displacement parameters (Å^2^)

**Table:**

	*x*	*y*	*z*	*U*_iso_*/*U*_eq_	
S	0.75906 (4)	0.44169 (9)	0.12214 (4)	0.05970 (15)	
C11	0.47717 (11)	0.9710 (2)	0.29664 (11)	0.0287 (3)	
H11	0.4771	1.0444	0.2271	0.034*	
O2	0.35347 (8)	1.10088 (15)	0.40682 (8)	0.0328 (2)	
O4	0.40208 (10)	0.43397 (19)	0.20264 (11)	0.0488 (3)	
H4	0.4583	0.5054	0.2205	0.073*	
C10	0.37095 (11)	0.8366 (2)	0.28037 (11)	0.0256 (3)	
H10	0.3907	0.7195	0.3274	0.031*	
O1	0.53049 (10)	1.22191 (19)	0.44127 (10)	0.0458 (3)	
C12	0.46117 (12)	1.1124 (2)	0.38823 (12)	0.0317 (3)	
O3	0.13476 (9)	0.9267 (2)	0.45208 (10)	0.0465 (3)	
C2	0.22047 (12)	0.8371 (2)	0.39912 (12)	0.0325 (3)	
H2	0.2684	0.7399	0.4466	0.039*	
N2	0.60890 (10)	0.70195 (19)	0.25656 (10)	0.0310 (3)	
C15	0.59162 (12)	0.8638 (2)	0.33252 (12)	0.0328 (3)	
H15A	0.6533	0.9583	0.3362	0.039*	
H15B	0.5962	0.8108	0.4075	0.039*	
C9	0.32885 (13)	0.7685 (2)	0.15807 (12)	0.0315 (3)	
H9A	0.3845	0.8096	0.1145	0.038*	
H9B	0.2576	0.8361	0.1277	0.038*	
C3	0.09931 (13)	0.7784 (2)	0.36578 (15)	0.0394 (4)	
C1	0.28464 (11)	0.9606 (2)	0.33070 (11)	0.0286 (3)	
H1	0.2315	1.0297	0.2711	0.034*	
C19	0.64040 (14)	0.7794 (3)	0.15414 (13)	0.0387 (3)	
H19A	0.7141	0.8447	0.1737	0.046*	
H19B	0.5841	0.8762	0.1201	0.046*	
C6	0.18706 (13)	0.4410 (2)	0.27405 (15)	0.0422 (4)	
H6	0.2559	0.4241	0.3251	0.051*	
C7	0.19578 (13)	0.4844 (2)	0.16970 (15)	0.0415 (4)	
C17	0.69958 (13)	0.5726 (3)	0.31802 (13)	0.0399 (4)	
H17A	0.6805	0.5356	0.3892	0.048*	
H17B	0.7711	0.6451	0.3343	0.048*	
C8	0.30887 (14)	0.5453 (2)	0.14119 (14)	0.0377 (3)	
H8	0.3042	0.5174	0.0612	0.045*	
C4	0.06950 (15)	0.5785 (3)	0.40637 (17)	0.0495 (4)	
H4A	−0.0082	0.5813	0.4192	0.059*	
H4B	0.1205	0.5488	0.4773	0.059*	
C16	0.71589 (16)	0.3889 (3)	0.25369 (16)	0.0496 (4)	
H16A	0.6447	0.3153	0.2384	0.059*	
H16B	0.7737	0.3070	0.2997	0.059*	
C18	0.64687 (16)	0.6178 (3)	0.07026 (14)	0.0498 (4)	
H18A	0.6606	0.6759	0.0012	0.060*	
H18B	0.5739	0.5497	0.0529	0.060*	
C13	0.02051 (14)	0.8583 (3)	0.26257 (18)	0.0547 (5)	
H13A	−0.0544	0.8785	0.2784	0.082*	
H13B	0.0157	0.7660	0.2019	0.082*	
H13C	0.0500	0.9812	0.2416	0.082*	
C5	0.07965 (16)	0.4157 (3)	0.32077 (19)	0.0537 (5)	
H5A	0.0811	0.2881	0.3570	0.064*	
H5B	0.0132	0.4197	0.2597	0.064*	
C14	0.09689 (18)	0.4951 (4)	0.06943 (19)	0.0679 (6)	
H14A	0.0262	0.4726	0.0938	0.102*	
H14B	0.1066	0.3965	0.0155	0.102*	
H14C	0.0952	0.6230	0.0355	0.102*	

### Atomic displacement parameters (Å^2^)

**Table:**

	*U*^11^	*U*^22^	*U*^33^	*U*^12^	*U*^13^	*U*^23^
S	0.0614 (3)	0.0736 (3)	0.0469 (2)	0.0211 (3)	0.0173 (2)	−0.0094 (3)
C11	0.0308 (7)	0.0301 (7)	0.0252 (6)	−0.0049 (5)	0.0051 (5)	0.0005 (5)
O2	0.0317 (5)	0.0312 (5)	0.0355 (5)	−0.0028 (4)	0.0070 (4)	−0.0086 (4)
O4	0.0411 (6)	0.0332 (6)	0.0703 (8)	0.0046 (5)	0.0068 (5)	−0.0056 (6)
C10	0.0270 (6)	0.0242 (6)	0.0249 (6)	−0.0015 (5)	0.0034 (5)	−0.0009 (5)
O1	0.0422 (6)	0.0481 (7)	0.0460 (6)	−0.0143 (5)	0.0061 (5)	−0.0175 (6)
C12	0.0325 (7)	0.0309 (7)	0.0310 (7)	−0.0039 (6)	0.0044 (5)	−0.0015 (6)
O3	0.0366 (5)	0.0539 (7)	0.0539 (7)	−0.0044 (6)	0.0205 (5)	−0.0164 (6)
C2	0.0283 (7)	0.0357 (7)	0.0339 (8)	0.0011 (6)	0.0072 (6)	−0.0026 (6)
N2	0.0291 (5)	0.0378 (6)	0.0271 (6)	0.0022 (5)	0.0077 (4)	0.0028 (5)
C15	0.0280 (7)	0.0415 (8)	0.0282 (7)	−0.0020 (6)	0.0039 (5)	−0.0038 (6)
C9	0.0353 (7)	0.0320 (7)	0.0259 (7)	−0.0036 (6)	0.0029 (5)	−0.0033 (6)
C3	0.0290 (7)	0.0384 (8)	0.0525 (10)	−0.0007 (6)	0.0123 (6)	−0.0048 (7)
C1	0.0277 (6)	0.0267 (7)	0.0294 (6)	0.0008 (6)	0.0008 (5)	−0.0027 (6)
C19	0.0427 (8)	0.0431 (9)	0.0325 (8)	−0.0002 (7)	0.0130 (6)	0.0051 (7)
C6	0.0388 (8)	0.0243 (7)	0.0621 (10)	−0.0001 (7)	0.0069 (7)	0.0012 (8)
C7	0.0381 (8)	0.0299 (8)	0.0531 (10)	−0.0042 (6)	0.0012 (7)	−0.0095 (7)
C17	0.0355 (8)	0.0521 (10)	0.0322 (8)	0.0092 (7)	0.0067 (6)	0.0046 (7)
C8	0.0419 (8)	0.0321 (8)	0.0373 (8)	−0.0015 (6)	0.0034 (6)	−0.0106 (6)
C4	0.0364 (9)	0.0519 (11)	0.0640 (11)	−0.0091 (8)	0.0191 (8)	0.0040 (9)
C16	0.0498 (9)	0.0484 (11)	0.0504 (10)	0.0141 (8)	0.0097 (8)	0.0056 (8)
C18	0.0586 (11)	0.0602 (11)	0.0312 (8)	0.0090 (9)	0.0107 (7)	−0.0017 (8)
C13	0.0335 (8)	0.0461 (10)	0.0776 (14)	0.0004 (8)	−0.0054 (8)	−0.0018 (9)
C5	0.0491 (9)	0.0350 (9)	0.0782 (13)	−0.0101 (8)	0.0157 (9)	0.0050 (9)
C14	0.0487 (10)	0.0836 (17)	0.0633 (13)	−0.0162 (11)	−0.0080 (9)	−0.0134 (11)

### Geometric parameters (Å, º)

**Table:**

S—C18	1.810 (2)	C1—H1	0.9800
S—C16	1.810 (2)	C19—C18	1.510 (3)
C11—C12	1.510 (2)	C19—H19A	0.9700
C11—C15	1.530 (2)	C19—H19B	0.9700
C11—C10	1.5410 (18)	C6—C7	1.325 (3)
C11—H11	0.9800	C6—C5	1.512 (2)
O2—C12	1.3500 (17)	C6—H6	0.9300
O2—C1	1.4592 (16)	C7—C14	1.516 (2)
O4—C8	1.424 (2)	C7—C8	1.516 (2)
O4—H4	0.8200	C17—C16	1.505 (3)
C10—C9	1.5396 (19)	C17—H17A	0.9700
C10—C1	1.5476 (19)	C17—H17B	0.9700
C10—H10	0.9800	C8—H8	0.9800
O1—C12	1.1983 (18)	C4—C5	1.539 (3)
O3—C2	1.4471 (18)	C4—H4A	0.9700
O3—C3	1.454 (2)	C4—H4B	0.9700
C2—C3	1.474 (2)	C16—H16A	0.9700
C2—C1	1.495 (2)	C16—H16B	0.9700
C2—H2	0.9800	C18—H18A	0.9700
N2—C19	1.4674 (19)	C18—H18B	0.9700
N2—C17	1.4735 (19)	C13—H13A	0.9600
N2—C15	1.4758 (19)	C13—H13B	0.9600
C15—H15A	0.9700	C13—H13C	0.9600
C15—H15B	0.9700	C5—H5A	0.9700
C9—C8	1.542 (2)	C5—H5B	0.9700
C9—H9A	0.9700	C14—H14A	0.9600
C9—H9B	0.9700	C14—H14B	0.9600
C3—C13	1.507 (3)	C14—H14C	0.9600
C3—C4	1.511 (3)		
			
C18—S—C16	96.67 (8)	C18—C19—H19B	109.3
C12—C11—C15	109.33 (11)	H19A—C19—H19B	108.0
C12—C11—C10	104.37 (11)	C7—C6—C5	128.33 (16)
C15—C11—C10	114.62 (12)	C7—C6—H6	115.8
C12—C11—H11	109.4	C5—C6—H6	115.8
C15—C11—H11	109.4	C6—C7—C14	125.47 (17)
C10—C11—H11	109.4	C6—C7—C8	121.62 (15)
C12—O2—C1	111.34 (10)	C14—C7—C8	112.74 (17)
C8—O4—H4	109.5	N2—C17—C16	112.94 (13)
C9—C10—C11	113.63 (12)	N2—C17—H17A	109.0
C9—C10—C1	115.55 (11)	C16—C17—H17A	109.0
C11—C10—C1	102.81 (11)	N2—C17—H17B	109.0
C9—C10—H10	108.2	C16—C17—H17B	109.0
C11—C10—H10	108.2	H17A—C17—H17B	107.8
C1—C10—H10	108.2	O4—C8—C7	111.77 (14)
O1—C12—O2	121.46 (14)	O4—C8—C9	111.68 (13)
O1—C12—C11	127.91 (14)	C7—C8—C9	111.03 (13)
O2—C12—C11	110.62 (11)	O4—C8—H8	107.4
C2—O3—C3	61.08 (10)	C7—C8—H8	107.4
O3—C2—C3	59.68 (10)	C9—C8—H8	107.4
O3—C2—C1	119.82 (13)	C3—C4—C5	111.69 (15)
C3—C2—C1	125.59 (13)	C3—C4—H4A	109.3
O3—C2—H2	113.7	C5—C4—H4A	109.3
C3—C2—H2	113.7	C3—C4—H4B	109.3
C1—C2—H2	113.7	C5—C4—H4B	109.3
C19—N2—C17	110.84 (12)	H4A—C4—H4B	107.9
C19—N2—C15	110.78 (12)	C17—C16—S	112.55 (13)
C17—N2—C15	107.95 (11)	C17—C16—H16A	109.1
N2—C15—C11	113.93 (11)	S—C16—H16A	109.1
N2—C15—H15A	108.8	C17—C16—H16B	109.1
C11—C15—H15A	108.8	S—C16—H16B	109.1
N2—C15—H15B	108.8	H16A—C16—H16B	107.8
C11—C15—H15B	108.8	C19—C18—S	112.36 (12)
H15A—C15—H15B	107.7	C19—C18—H18A	109.1
C10—C9—C8	115.79 (13)	S—C18—H18A	109.1
C10—C9—H9A	108.3	C19—C18—H18B	109.1
C8—C9—H9A	108.3	S—C18—H18B	109.1
C10—C9—H9B	108.3	H18A—C18—H18B	107.9
C8—C9—H9B	108.3	C3—C13—H13A	109.5
H9A—C9—H9B	107.4	C3—C13—H13B	109.5
O3—C3—C2	59.24 (9)	H13A—C13—H13B	109.5
O3—C3—C13	113.05 (14)	C3—C13—H13C	109.5
C2—C3—C13	122.86 (15)	H13A—C13—H13C	109.5
O3—C3—C4	116.16 (15)	H13B—C13—H13C	109.5
C2—C3—C4	115.81 (15)	C6—C5—C4	111.07 (15)
C13—C3—C4	116.52 (15)	C6—C5—H5A	109.4
O2—C1—C2	107.44 (11)	C4—C5—H5A	109.4
O2—C1—C10	105.77 (10)	C6—C5—H5B	109.4
C2—C1—C10	111.70 (12)	C4—C5—H5B	109.4
O2—C1—H1	110.6	H5A—C5—H5B	108.0
C2—C1—H1	110.6	C7—C14—H14A	109.5
C10—C1—H1	110.6	C7—C14—H14B	109.5
N2—C19—C18	111.59 (15)	H14A—C14—H14B	109.5
N2—C19—H19A	109.3	C7—C14—H14C	109.5
C18—C19—H19A	109.3	H14A—C14—H14C	109.5
N2—C19—H19B	109.3	H14B—C14—H14C	109.5

### Hydrogen-bond geometry (Å, º)

**Table:**

*D*—H···*A*	*D*—H	H···*A*	*D*···*A*	*D*—H···*A*
O4—H4···N2	0.82	2.21	3.0278 (18)	176
C2—H2···O1^i^	0.98	2.51	3.2948 (19)	137
C6—H6···O2^ii^	0.93	2.59	3.2526 (19)	129

Symmetry codes: (i) −*x*+1, *y*−1/2, −*z*+1; (ii) *x*, *y*−1, *z*.
