# Supplementary figures and images for: Crystal structure of (1S,2R,4R,9S,11S,12R)-9α-hy­droxy-4,8-dimethyl-12-[(thio­morpholin-4-yl)meth­yl]-3,14-dioxatri­cyclo­[9.3.0.02,4]tetra­dec-7-en-13-one
Source: Acta Crystallogr E Crystallogr Commun. 2015 Jan 31;71(Pt 2):o140–1. doi: 10.1107/S205698901500170X (PMC4384556; doi:10.1107/S205698901500170X)

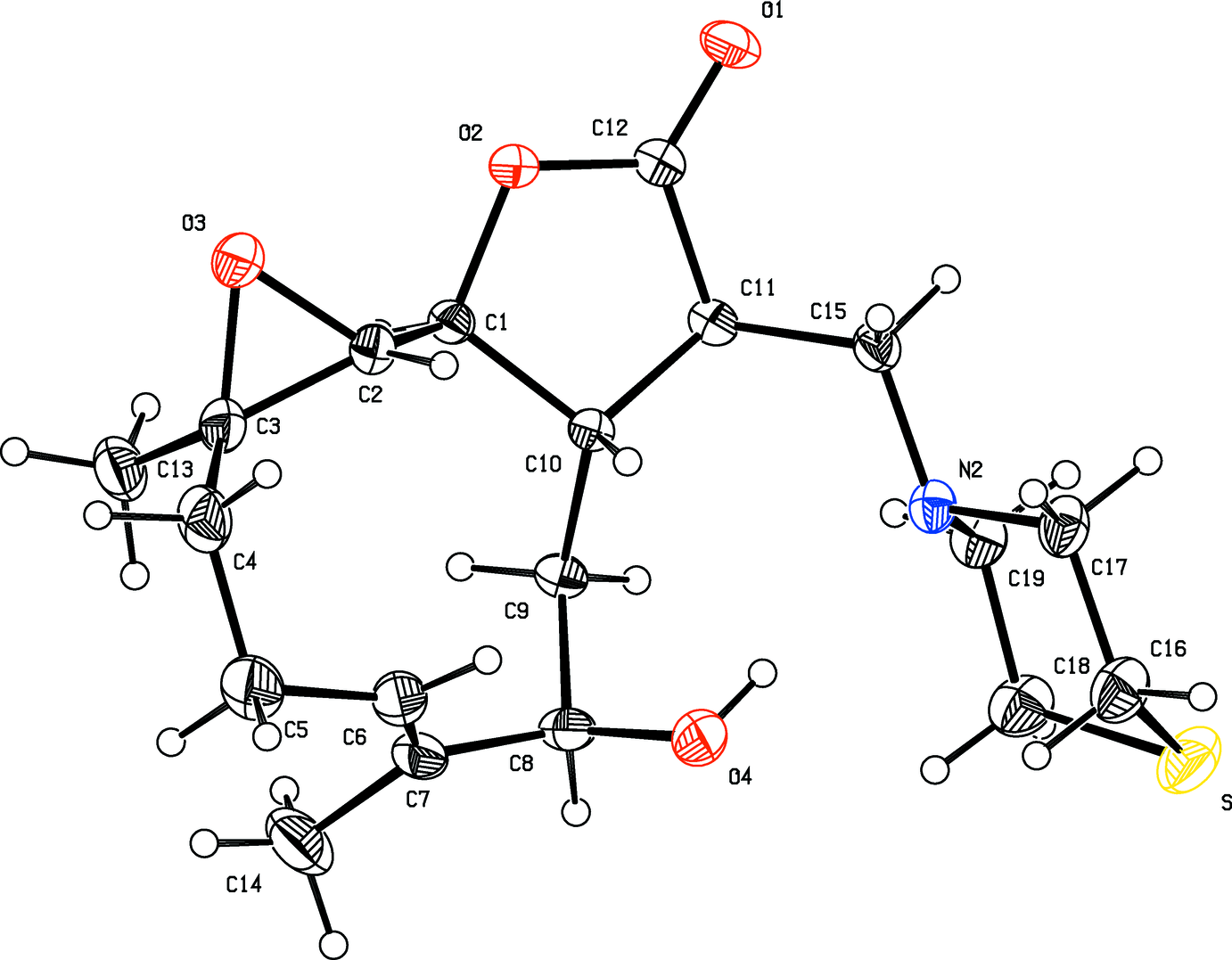

Supplement: Supplementary file 4 [file e-71-0o140-fig1.tif]

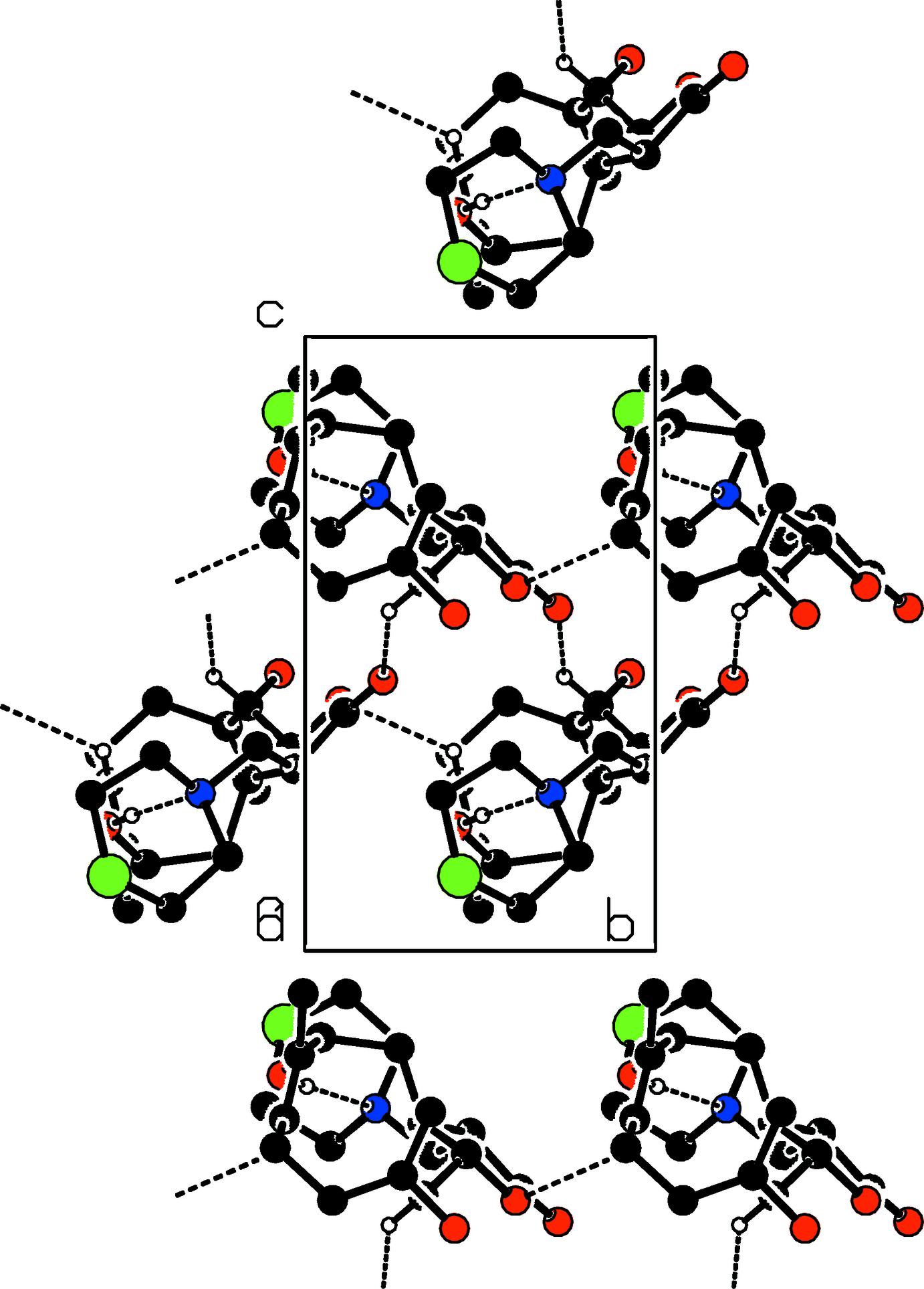

Supplement: Supplementary file 5 [file e-71-0o140-fig2.tif]
